# Bedside management of a knotted Swan-Ganz catheter – A case report and literature review

**DOI:** 10.1016/j.ijscr.2025.111013

**Published:** 2025-02-04

**Authors:** Bassam Osman, Bassel Hafez, Aya El Madani, Vahe S. Panossian, Olga Dirany, Pierre Sfeir

**Affiliations:** a Department of Surgery, American University of Beirut Medical Center, Beirut, Lebanon; b Division of Cardiothoracic Surgery, Department of Surgery, American University of Beirut Medical Center, Beirut, Lebanon

**Keywords:** Pulmonary artery catheter, Swan-Ganz catheter, Knot, Complication

## Abstract

**Introduction and importance:**

Pulmonary artery catheters use remain invaluable in continuous invasive hemodynamic monitoring for patients with severe cardiopulmonary dysfunction and those undergoing major cardiac surgeries. It detects cardiac dysfunction and guides treatment decisions. Its utilization has declined due to common complications associated with its insertion. This article highlights a rare, rather an important complication of pulmonary artery catheter knotting and reviews techniques for its management. Prompt recognition of this rare complication by the clinicians allow immediate intervention minimizing morbidity and optimizing the outcomes. This manuscript follows the SCARE guidelines.

**Case presentation:**

A case of a 61-year-old man who was initially admitted to the cardiothoracic unit for mitral valve replacement for symptomatic severe mitral regurgitation secondary to a bi-leaflet prolapse. Intra-operatively, a pulmonary artery catheter was inserted for invasive continuous hemodynamic monitoring. It was noted on a routine postoperative chest X-ray coiling of the pulmonary artery catheter in the right atrium. The catheter was not repositioned. On postoperative day one, significant resistance was encountered while removing the catheter. A chest x-ray was done and showed a knotted catheter in the superior vena cava. After contingency planning, bedside removal was opted as the preferred management approach. The catheter was carefully pulled out to tighten the knot and decrease its diameter, allowing successful extraction through the insertion site at the neck.

**Clinical discussion:**

Pulmonary artery catheter utilization remains essential; however its insertion is prone to complications, which includes pulmonary artery catheter knotting a rare but an important complication that warrants immediate recognition and management. Numerous approaches for the management of knotted catheter were described in the literature. Bedside removal of the knotted catheter may be considered if the knot is simple and more proximal. This approach carries the risk of venous injury and hematoma formation, thus thorough planning and preparation are needed to avoid complications. An endovascular approach is opted in more complex cases and has largely supplanted surgical methods. Innovative methods were described in the literature and include the introduction of guide wires or specialized catheters to untangle knots under fluoroscopic guidance. Retrieval baskets may also be used. Surgery is reserved for complex cases, when endovascular attempts fail.

**Conclusion:**

While pulmonary artery catheter use provides significant benefits, its use demands meticulous planning and preparation to avoid complications. Catheter knotting requires immediate attention. If bedside maneuvers fail, endovascular or surgical approaches may be necessary.

## Highlights

• This case report presents the management of a rare complication, catheter knotting, during the use of a pulmonary artery catheter (PAC), highlighting its clinical implications and potential risks.
• We successfully managed a knotted Swan-Ganz catheter at the bedside, emphasizing techniques to safely address simple and localized knots in stable patients while preparing for emergent intervention if needed.
• The report reviews bedside, endovascular, and surgical options for knotted catheter removal, providing a comprehensive guide for clinicians to tailor management based on the knot's complexity and patient stability.

## 1 Introduction

Pulmonary artery catheters (PACs), often called Swan-Ganz catheters, are commonly used for advanced continuous hemodynamic monitoring in critically ill patients. Their perioperative use offers pivotal information regarding cardiac performance such as detecting right ventricular dysfunction. Moreover, the right ventricular pressure waveform guides fluid management, administration of cardio-tonic agents, and cardiac pacing. Their use remains invaluable in high-risk complex cardiac surgeries and cases of significant cardiopulmonary dysfunction [1]. Current guidelines recommend selective and tailored utility of the catheter based on patient risks and clinical context due to their potential complications [[2], [3], [4], [5]].

PAC insertion is a minimally invasive procedure. Its insertion involves advancing the catheter through the right internal jugular vein into the pulmonary artery. However, their routine use for invasive hemodynamic monitoring in critically ill patients has decreased. Their complications often outweigh their benefit [3,4,[6], [7], [8], [9], [10], [11], [12], [13]]. One notable complication is catheter knotting. It is a rare but challenging complication that may result in procedural difficulties and morbidity [14].

This study presents a case of Swan-Ganz catheter knotting. It outlines the bedside management for its removal, reviews the existing literature, and highlights different management techniques to offer applicable and practical insights for healthcare workers.

## 2 Case

**Fig. 1:**
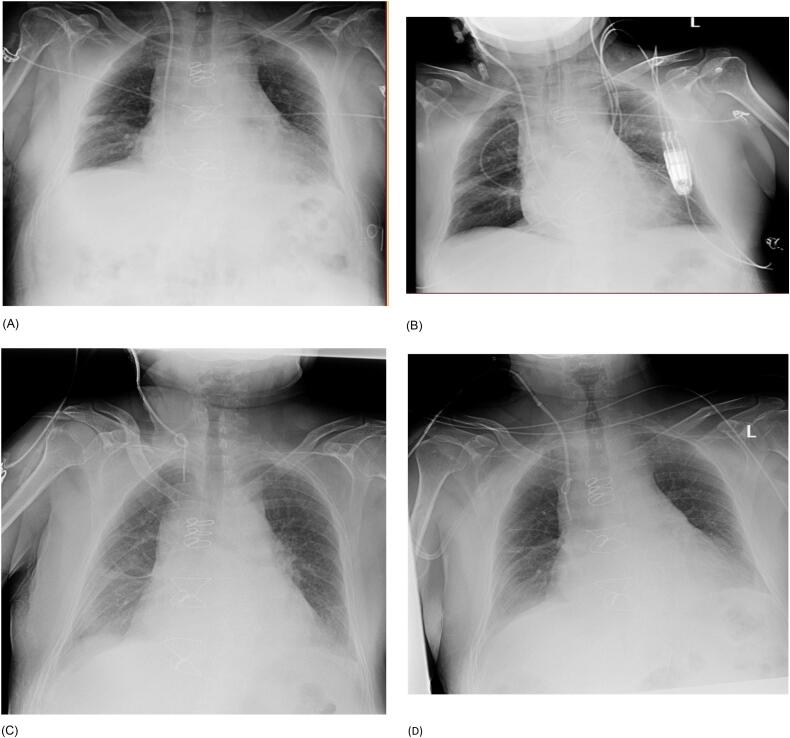
A: Right Internal Jugular Swan-Ganz catheter is seen coiled inside to the right atrium and terminating in the right ventricle. B: The right internal jugular Swan-Ganz catheter is still seen coiling and terminating inside the right atrium. C: Knotted Swan-Ganz catheter observed in the superior vena cava. D: Knotted catheter is withdrawn to the level of the internal jugular vein.

**Fig. 2:**
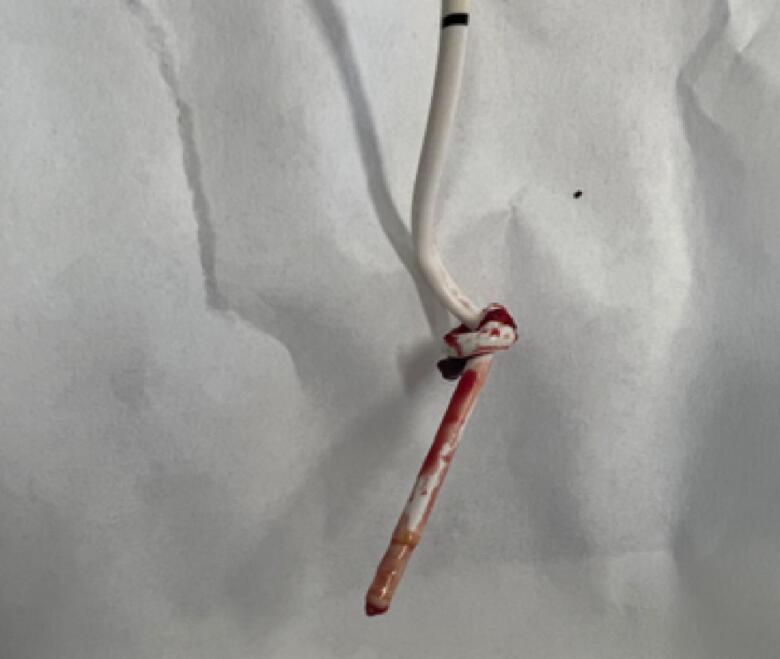
Removed catheter.

A case of a 61-year-old man, smoker, with a history of dyslipidemia and hypertension presented for three months history of worsening dyspnea on exertion. A transthoracic echocardiogram showed normal left ventricular systolic function with an estimated ejection fraction of 65–69 % and prolapse of A2 and P3 segments of the mitral valve with severe mitral regurgitation. An elective coronary angiography performed showed normal coronaries. He was admitted to the cardiac care unit for mitral valve replacement. Preoperative assessment revealed a revised cardiac risk index 1 and major cardiac event score 6 %, meaning he is intermediate risk for major cardiac events. Preoperatively, a right internal jugular vein central venous catheter was inserted under ultrasound guidance, then a non-oximetric, 5 lm Arrow® thermodilution 7.5 Fr × 110 cm pulmonary artery catheter was placed through the introducer. The placement was confirmed by pressure tracing changes and the insertion was well tolerated. He underwent a mitral valve replacement with a with a 29 mm Hancock II® porcine valve, intraoperative transesophageal echocardiogram confirmed proper positioning and function without any paravalvular leak. The operation was uneventful, he remained hemodynamically stable. He tolerated the procedure well and was then transferred to the cardiac surgery unit (CSU) in stable condition for 24 h monitoring. A routine post-operative chest X-ray revealed a coiled PAC in the right atrium (Fig. 1, (A)). The catheter was left in place without repositioning as it was functional providing adequate hemodynamic measurements, was not causing arrhythmias and its use was temporary. Overnight, the patient remained hemodynamically stable and was resuscitated when needed. On postoperative day one, he was stable and was ready for transfer to the coronary care unit (CCU). A morning routine chest X-ray again demonstrated a coiled catheter in the right atrium (Fig. 1, (B)). The Swan-Ganz catheters are usually removed prior to transfer if no persistent indication for invasive monitoring exists in accordance with the institutional protocol. During removal, the catheter was withdrawn over the cordis with success until resistance was encountered after 5 cm of withdrawal. A chest X-ray revealed the Swan-Ganz catheter was knotted within the superior vena cava (Fig. 1, (C)). The cordis was removed completely. The catheter was pulled with minimal resistance for an additional 10 cm until further withdrawal was halted due to major resistance. Another chest X-ray was done, and it confirmed that the knot was stuck in the internal jugular (Fig. 1, (D)). The vascular surgery service was immediately consulted to evaluate the possibility of surgical or endovascular removal of the catheter. Since the knot was at the tip of the catheter and resided in the neck, a decision was made to apply a steady withdrawal force to allow the knot to tighten and decrease in size, facilitating its passage through the puncture site. As a contingency, the operating room team was informed to prepare for a potential cut down over the jugular vein for venotomy and extraction. However, after steady pulling force was applied to the catheter, it was successfully extracted. Compression was maintained at the insertion site for 20 min (Fig. 2). Following this, the insertion site was dry, and the neck was clear of hematomas. The patient remained stable and was transferred to the coronary care unit. The following day, the patient remained stable with a normal neck examination.

The patient had a routine seamless postoperative course and was discharged home on postoperative day five. He was doing well on follow-up after three weeks.

## 3 Discussion

Various complications associated with PACs use have been reported in literature [15]. These complications can arise during initial venous access, such as injury to nearby arteries or nerves, hemorrhage, pneumothorax, or air embolism [[16], [17], [18], [19], [20]]. Additional complications may include arrhythmias during catheter placement [21], and post-operative complications: pulmonary embolism, valvular injury, infection, catheter knotting, and pulmonary artery rupture [15,22,23]. The mortality rates related to the routine use of Swan-Ganz catheters have been reported to be between 0.02 % and 1.5% [[24], [25], [26]].

The most common uses of PACs are for assessing the volume status in shock, pulmonary hypertension, and patients with severe cardiopulmonary disease undergoing surgery [7,[27], [28], [29], [30]]. Their use has been subjected to controversy and debate. Studies have shown no benefit in survival or hospital length of stay in critically ill patients who have PACs [[7], [8], [9], [10], [11], [12]]. Thus, their use has declined [13], with societal guidelines no longer supporting their routine use [[2], [3], [4], [5]]. Although catheter knotting is rare, it accounts for more than two-thirds of all reported cases of intravascular device knot formation. Knotting is mainly attributed to the lack of fluoroscopic guidance during placement, the catheter's thin wall, and soft construction, which mostly occurs at the level of the right atrium or ventricle [14].

Numerous approaches for the management of knotted catheters were described, varying between conservative and invasive. 62 % of the knotted intravascular catheters were withdrawn successfully with different interventional radiological techniques. In 32 % of the patients, surgical removal was deemed appropriate. However, in almost 6 % of the cases where the clinical condition was critical prohibiting any surgical intervention, the knotted catheter was left in place which increases the likelihood of thrombosis [14].

Multiple bedside approaches have been described in the literature for managing knotted intravascular catheters. A common approach entails applying a steady, gentle pulling force to tighten the knot and reduce its diameter allowing removal through its insertion site [31]. However, this method may carry the risk of venous trauma and hematoma formation [14]. A safer approach involves making a small skin incision once the knot reaches the surface, then removing the catheter under vision through a venotomy [32]. Although these techniques can be effective, they require skilled medical professionals, thorough planning, and meticulous preparation to avoid life-threatening complications.

Endovascular approaches have also been of utility for the management of the PACs knots. Under fluoroscopic guidance, a straight 0.018-inch rigid guide wire can be introduced into the PAC's distal port and advanced into the knot to straighten and unwind it. The PAC can be gently pulled out afterward without the need for vascular access [33,34]. A larger more flexible guide wire (0.038 in.) can be used for larger and more complex knots. The larger diameter provides more strength to untangle the knot, and the flexibility allows better manipulation of the knot [35]

Other innovative methods are present in the literature and include using a retrieval basket. A Dornier® retrieval basket can be introduced through the right femoral vein to the knotted catheter under fluoroscopy guidance. With gentle manipulation, the catheter can be pulled to the femoral vein level. Then it can be extracted by femoral vein cutdown [36]. A different technique highlighted in the literature includes introducing a percutaneous sheath into the femoral vein under fluoroscopy, followed by inserting a guide wire and advancing into the loop of the knot of the PAC. A 5 F angioplasty catheter with a 10 mm diameter and a 4 mm balloon can be passed over the guide wire, the knot should be positioned in the middle of the balloon. The balloon is then inflated releasing the knot and the catheter can then be pulled out [37].

In other cases, a 7 F Teflon® catheter was embedded through the femoral vein under fluoroscopic guidance and a Cook® deflecting wire utilized to snare the knot. By applying opposing gentle traction, the knot would untie. The removal of the catheter may be assisted by a venotomy [38].

A loop-snare catheter may be advanced through the femoral vein under fluoroscopic guidance, grasping the knotted catheter and removing it through a venotomy [14,39,40].

Another method described passing a sheath and guide wire through the knot and inflating the balloon to loosen the knot, then advancing the loop-snare catheter grasping the distal end of the PAC untying the knot and removing it percutaneously. Rotational angiography allows visualizing the proper positioning of the snare to avoid further knotting [41].

Another study described inserting an 8 F Mullin® sheath through the femoral vein and was guided via the 0.35-inch J-wire through the loop of the knot unraveling it by applying traction [42].

The choice of surgical or endovascular approach depends on the size, location, and structural complexity of the knot.

Surgical approaches are generally reserved for larger knots with many loops or intracardiac involvement. A study described a simple surgical intervention by performing a simple transverse jugular venotomy and removing the introducer sheath and the catheter. Endovascular removal might be attempted first in such cases [43]. Another study demonstrated simple jugular venotomy [44]. One study demonstrated a case of surgical management of a knotted catheter in the right atrium. A right atrium purse-string incision was made, the knot was cut, and the catheter was successfully removed [45]. In another study, the PAC had multiple knots at the level of the right ventricle. The patient was continuously bleeding. Thus, a surgical approach was chosen to control the bleeding and remove the catheter by employing a tobacco pouch in the right atrium [46]. Another study reported a case of intraoperative PAC knotting in the superior vena cava. In this case, the catheter was directed into the azygous vein. A venotomy was performed to section and remove the catheter. The azygous vein was then ligated, and the proximal portion of the catheter was extracted through the neck using an introducer [47]. A study demonstrated a knotted PAC that couldn't be retrieved after multiple attempts by intervention radiology. It was noted that the knot was stuck at the tricuspid valve. The patient was operated on, a cardiopulmonary bypass was performed, the right atrium was explored, and the knot was formed around the chordal structure of the tricuspid valve anterior leaflet. The knot was released, and the catheter was withdrawn [48].

A combined endovascular and surgical approach has also been reported. To avoid major thoracic open surgery, a knotted catheter in the subclavian vein was relocated under fluoroscopic guidance to the femoral vein. The catheter was then removed by femoral vein venotomy [49].

In the case we described, bedside management of the knotted Swan-Ganz Catheter was appropriate due to several factors. The knot was simple and localized in the superior vena cava. Moreover, the patient was hemodynamically stable. Adding to this, the operating room was prepared for immediate intervention if needed. Endovascular or surgical approaches were likely to be considered if the knot was more complex or located more proximally. While we achieved successful removal, this method carried the risk of venous trauma and hematoma formation, which would've necessitated surgical exploration for control of bleed and vascular repair.

## 4 Conclusion

PACs add benefit to the management of critically ill patients especially those with severe cardiopulmonary dysfunction and those undergoing major cardiac surgery. Nonetheless their use is associated with possible complications such as knotting in a central vein. These adverse events require immediate attention. Understanding the risks and applying careful catheter manipulation during insertion and removal can help minimize such complications. If catheter knotting cannot be resolved through bedside maneuvers endovascular or surgical approaches can be considered.

## Author contribution

Pierre Sfeir was the primary surgeon and supervisor. BO, BH, AEM and VP revised the literature and edited the manuscript. OD was involved in perioperative management.

## Consent

Written informed consent was obtained from the patient for publication and any accompanying images. A copy of the written consent is available for review by the Editor-in-Chief of this journal on request.

## Ethical approval

Ethical approval was given for this study by the Institutional Review Board at the American University of Beirut.

## Guarantor

Pierre Sfeir.

## Research registration number

None.

## Funding

No funding was provided for this study.

## Conflict of interest statement

The authors have no competing interests.

## Data Availability

Data can be obtained by contacting corresponding author.

## References

[1] Sohrabi C., Mathew G., Maria N., Kerwan A., Franchi T., Agha R.A. (2023). The SCARE 2023 guideline: updating consensus Surgical CAse REport (SCARE) guidelines. Int. J. Surg. Lond. Engl..

[2] NA. (2003). Practice guidelines for pulmonary artery catheterization. Anesthesiology.

[3] Fleisher L.A., Fleischmann K.E., Auerbach A.D. (2014). 2014 ACC/AHA Guideline on Perioperative Cardiovascular Evaluation and Management of Patients Undergoing Noncardiac Surgery: executive summary: a report of the American College of Cardiology/American Heart Association Task Force on Practice Guidelines. Circulation.

[4] Mueller H., Chatterjee K., Davis K. (1998). Present use of bedside right heart catheterization in patients with cardiac disease11This Expert Consensus Document was approved by the American College of Cardiology Board of Trustees in March 1998.22Address for reprints: Educational Services, American College of Cardiology, 9111 Old Georgetown Road, Bethesda, Maryland 20814-1699. J. Am. Coll. Cardiol..

[5] (2014). 2014 ESC/ESA Guidelines on non-cardiac surgery: cardiovascular assessment and management: the Joint Task Force on non-cardiac surgery: cardiovascular assessment and management of the European Society of Cardiology (ESC) and the European Society of Anaesthesiology (ESA). Eur. Heart J..

[6] (2014). 2014 ESC/ESA Guidelines on non-cardiac surgery: cardiovascular assessment and management: the Joint Task Force on non-cardiac surgery: cardiovascular assessment and management of the European Society of Cardiology (ESC) and the European Society of Anaesthesiology (ESA). Eur. Heart J..

[7] Richard C. (2003). Early use of the pulmonary artery catheter and outcomes in patients with shock and acute respiratory distress syndrome a randomized controlled trial. JAMA.

[8] Connors A.F. (1996). The effectiveness of right heart catheterization in the initial care of critically III patients. JAMA.

[9] Sandham J.D., Hull R.D., Brant R.F. (2003). A randomized, controlled trial of the use of pulmonary-artery catheters in high-risk surgical patients. N. Engl. J. Med..

[10] Yu D.T., Platt R., Lanken P.N. (2003). Relationship of pulmonary artery catheter use to mortality and resource utilization in patients with severe sepsis*. Crit. Care Med..

[11] Shah M.R., Hasselblad V., Stevenson L.W. (2005). Impact of the pulmonary artery catheter in critically ill patients: meta-analysis of randomized clinical trials. JAMA.

[12] Harvey S., Harrison D.A., Singer M. (2005). Assessment of the clinical effectiveness of pulmonary artery catheters in management of patients in intensive care (PAC-Man): a randomised controlled trial. Lancet.

[13] Wiener R.S., Welch H.G. (2007). Trends in the use of the pulmonary artery catheter in the United States, 1993-2004. JAMA.

[14] Karanikas I.D., Polychronidis A., Vrachatis A., Arvanitis D.P., Simopoulos C.E., Lazarides M.K. (2002). Removal of knotted intravascular devices. Case report and review of the literature. Eur. J. Vasc. Endovasc. Surg..

[15] Lopes M.C., Cleva R.D., Zilberstein B., Gama-Rodrigues J.J. (2004). Pulmonary artery catheter complications: report on a case of a knot accident and literature review. Rev. Hosp. Clin..

[16] Puri V.K., Carlson R.W., Bander J.J., Weil A.H. (1980). Complications of vascular catheterization in the critically ill: a prospective study. Crit. Care Med..

[17] Mansfield P.F., Hohn D.C., Fornage B.D., Gregurich M.A., Ota D.M. (1994). Complications and failures of subclavian-vein catheterization. N. Engl. J. Med..

[18] Elliott C.G., Zimmerman G.A., Clemmer T.P. (1979). Complications of pulmonary artery catheterization in the care of critically III patients. Chest.

[19] Sise M.J., Hollingsworth P., Brimm J.E., Peters R.M., Virgilio R.W., Shackford S.R. (1981). Complications of the flow-directed pulmonary-artery catheter: a prospective analysis in 219 patients. Crit. Care Med..

[20] Dee Boyd K., Thomas S.J., Gold J., Boyd A.D. (1983). A prospective study of complications of pulmonary artery catheterizations in 500 consecutive patients. Chest.

[21] Sprung C.L., Jacobs L.J., Caralis P.V., Karpf M. (1981). Ventricular arrhythmias during Swan-Ganz catheterization of the critically III. Chest.

[22] Pinilla J.C., Ross D.F., Martin T., Crump H. (1983). Study of the incidence of intravascular catheter infection and associated septicemia in critically ill patients. Crit. Care Med..

[23] Bossert T., Gummert J.F., Bittner H.B. (2006). Swan-Ganz catheter-induced severe complications in cardiac surgery: right ventricular perforation, knotting, and rupture of a pulmonary artery. J. Card. Surg..

[24] Ginosar Y., Sprung C.L. (1996). The SWAN-GANZ catheter. Crit. Care Clin..

[25] (1997). Pulmonary artery catheter consensus conference: consensus statement. Crit. Care Med..

[26] Eshkevari L., Baker B.M. (2007). Occurrence and removal of a knotted pulmonary artery catheter: a case report. AANA J..

[27] Cohen M.G., Kelly R.V., Kong D.F. (2005). Pulmonary artery catheterization in acute coronary syndromes: insights from the GUSTO IIb and GUSTO III trials. Am. J. Med..

[28] Pandey A., Khera R., Kumar N., Golwala H., Girotra S., Fonarow G.C. (2016). Use of pulmonary artery catheterization in US patients with heart failure, 2001-2012. JAMA Intern. Med..

[29] Rapoport J. (2000). Patient characteristics and ICU organizational factors that influence frequency of pulmonary artery catheterization. JAMA.

[30] Koo K.K.Y., Sun J.C.J., Zhou Q. (2011). Pulmonary artery catheters: evolving rates and reasons for use*. Crit. Care Med..

[31] Starzyk L., Yao E., Roche-Nagel G., Wasowicz M. (2016). Snaring swans: intraoperative knotting of pulmonary artery catheters. Anaesth. Intensive Ther..

[32] Kumar S.P. (1980). Removal of a knotted flow-directed catheter by a nonsurgical method. Ann. Intern. Med..

[33] Egbuche O., Nwagbara K., Mezue K.N., Abe T., Nwokike S. (2021). Transvenous retrieval of a pulmonary artery catheter knot around pacing leads: a case report. Cardiovasc. Revasc. Med..

[34] Hong B., Park H., Yoon Y., Jung C., Kim Y.H., Lim C.S. (2018). Interventional removal of knotted pulmonary artery catheter: a case report. J. Intensive Care Med..

[35] Mond H.G., Clark D.W., Nesbitt S.J., Schlant R.C. (1975). A technique for unknotting an intracardiac flow-directed balloon catheter. Chest.

[36] Hood S., McAlpine H.M., Davidson J.A.H. (1997). Successful retrieval of a knotted pulmonary artery catheter trapped in the right ventricle using a Dormier basket. Scott. Med. J..

[37] Tan C., Bristow P.J., Segal P., Bell R.J. (1997). A technique to remove knotted pulmonary artery catheters. Anaesth. Intensive Care.

[38] Cho Tisnado J., Beachley M., Vines F., Alford W. (1983). Percutaneous unknotting of intravascular catheters and retrieval of catheter fragments. Am. J. Roentgenol..

[39] Ltaief Z., Qanadli S.D., Eckert P., Ben-Hamouda N. (2020). Video fluoroscopy for pulmonary artery catheter insertion in high-risk situation of knotting or misplacement. Eur. Rev. Med. Pharmacol. Sci..

[40] Kalińczuk Ł., Chmielak Z., Dębski A. (2016). Percutaneous retrieval of centrally embolized fragments of central venous access devices or knotted Swan-Ganz catheters. Clinical report of 14 retrievals with detailed angiographic analysis and review of procedural aspects. pwki.

[41] Mima H., Enomoto S., Tamaki Y., Miyake M., Kondo H., Tamura T. (2022). Percutaneous removal of a knotted Swan–Ganz catheter. Cardiovasc. Interv. Ther..

[42] Rahim S.A., Franke R., Mathew V. (2009). Removal of a knotted Swan-Ganz catheter. J. Am. Coll. Cardiol..

[43] Wang N.N., Hatzakorzian R., Carvalho G., Waters P. (2015). Entrapment of a pulmonary artery catheter inside a knotted percutaneous sheath introducer. Can. J. Anesth./J. Can. Anesth..

[44] Bagul N.B., Menon N.J., Pathak R., Platts A., Hamilton G. (2005). Knot in the cava—an unusual complication of Swan–Ganz catheters. Eur. J. Vasc. Endovasc. Surg..

[45] Samarkandi A.H., Al Satli R., Maher S., Al Watidy A.F. (1995). Surgical removal of a knotted pulmonary artery catheter. Ann. Saudi Med..

[46] Bonet N.A., Samper F.O., Loro Represa J.M., Guillén R.V. (2010). Multiple knotting of a Swan-Ganz catheter. Revista Española de Cardiología (English Edition)..

[47] De Jesus Peixoto Camargo J., Marcantonio Camargo S., Noguchi Machuca T., Wagner P. (2008). Intraoperative removal of a knotted Swan-Ganz catheter during lung transplantation. Interact. Cardiovasc. Thorac. Surg..

[48] Perez d’Empaire P., Derzi S., Latter D., Tousignant C. (2019). Pulmonary artery catheter knotted in the tricuspid valve apparatus requiring surgery with cardiopulmonary bypass: a case report. A&A Practice.

[49] Papakostas J.C., Papadopoulos L.S.N., Arnaoutoglou H.M., Karahaliou A., Matsagas M.I. (2008). Transfemoral removal of a knotted Swan–Ganz catheter. Can. J. Surg..

